# Advances in immunotherapy for the treatment of glioblastoma

**DOI:** 10.1007/s11060-016-2299-2

**Published:** 2016-10-14

**Authors:** Amanda Tivnan, Tatjana Heilinger, Ed C. Lavelle, Jochen H. M. Prehn

**Affiliations:** 1 Department of Physiology and Medical Physics and RCSI Centre for Systems Medicine, Royal College of Surgeons in Ireland, 123 St. Stephen’s Green, Dublin 2, Ireland; 2 IMC Fachhochschule Krems, University of Applied Sciences, Piaristengasse 1, 3500 Krems, Austria; 3 Adjuvant Research Group, School of Biochemistry and Immunology, Trinity Biomedical Sciences Institute, Trinity College Dublin, Dublin, D02 PN40 Ireland; 4 Centre for Research on Adaptive Nanostructures and Nanodevices (CRANN), Trinity College Dublin, Dublin 2, D02 PN40 Ireland; 5 Advanced Materials Bio-Engineering Research Centre (AMBER), Trinity College Dublin, Dublin 2, D02 PN40 Ireland

**Keywords:** Glioblastoma, Immunotherapy, Brain cancer, Vaccines

## Abstract

Glioblastoma (GBM) is an aggressive brain tumour, associated with extremely poor prognosis and although there have been therapeutic advances, treatment options remain limited. This review focuses on the use of immunotherapy, harnessing the power of the host’s immune system to reject cancer cells. Key challenges in glioma specific immunotherapy as with many other cancers are the limited immunogenicity of the cancer cells and the immunosuppressive environment of the tumour. Although specific antigens have been identified in several cancers; brain tumours, such as GBM, are considered poorly immunogenic. However, as detailed in this review, strategies aimed at circumventing these challenges are showing promise for GBM treatment; including identification of glioma specific antigens and endogenous immune cell activation in an attempt to overcome the immunosuppressive environment which is associated with GBM tumours. An up-to-date summary of current Phase I/II and ongoing Phase III GBM immunotherapy clinical trials is provided in addition to insights into promising preclinical approaches which are focused predominantly on increased induction of Type 1 helper T cell (T_h_1) immune responses within patients.

## Introduction

Glioblastoma (GBM), a highly aggressive solid neoplasm with an average 5 year survival rate of <5 %, is the most lethal form of brain tumour (http://www.braintumourresearch.org/our-reports). Median survival rates for GBM patients have not changed significantly with current standard of care, involving tumour resection followed by radiotherapy (RT) with adjunct and concomitant temozolomide (TMZ). However this has limited impact, with GBM recurrence at distal sites within 7 months [1] with adjunct chemotherapy being ineffective at stopping tumour progression and morbidity. In this regard, novel GBM treatments are being investigated including immunotherapy.

## Tumour microenvironment

GBM tumours are inherently heterogeneous, each cell type contributing towards disease pathogenesis. Although the role of stem-like cells has been extensively evaluated, their contribution to relapse, chemo- and radio-therapy resistance [2] and the role of vascular cells such as microglia, peripheral immune and neural processor cells; in the generation of a specific niche within which GBM cells can evade immune detection is a topic of ongoing research. A detailed understanding of the supportive role that the microenvironment plays in GBM is critical to the design of effective immunotherapeutic strategies. Glioma histology shows that >30 % of GBM tumours are composed of infiltrating microglia [3] with active recruitment of peripheral macrophages [4]; for the purpose of this review we have focused on the contribution which microglia play in GBM immune evasion.

The secretion of immunomodulatory cytokines from GBM cells, including interleukins 10 (IL-10), 4 (IL-4) and 6 (IL-6), and particularly, tumour growth factor-beta (TGF-β) in addition to prostaglandin E2 [5] can supress microglia activation [6]. This, in combination with reduced levels of major histocompatibility complex (MHC)II expression on GBM microglia significantly contributes to immune evasion. Microglia have been show to increase GBM cell migration and invasion through interaction with membrane type I metalloproteinases (MMPs) and secretion of matrix-degrading enzymes [7]. Inhibition of TGF-β/Smads signalling restores immune surveillance in glioma models [8] inhibiting proliferation through platelet-derived growth factor-β (PDGF-β) and microRNA-182. Additionally, invasiveness is inhibited via microRNA-182 and -10 and matrix metalloproteinases (MMP), with angiogenesis inhibition via vascular endothelial growth factor (VEGF), Insulin-like growth factor-binding protein 7 (IGFBP7), and c-Jun N-terminal kinases (JNK). Inhibition of the TGF-β/Smads signalling pathway restores immunosurveillance by activating natural killer (NK) cells, cytotoxic T lymphocytes (CTL) and dendritic cells (DC), and by downregulating T regulatory (Treg) cells. TGF-β inhibition also reduces glioma stem-like cell (GSC) stemness via Leukaemia inhibitory factor (LIF), Sox4-Sox2, and inhibitor of DNA binding 1–3 (Id1–Id3) [9]. Notably, in clinical trials, toxicity is significant (reviewed by Han et al. [9]) however a Phase IIb trial using TGF-β antisense (Trabedersen) showed promise [10] but further studies have not progressed to date.

Although oversimplification, macrophage, or microglia, activation can be categorised as M1-activation which is promoted by interferon gamma and contributes to T_h_1 responses and M2-activation which can be promoted by IL-4 and IL-13 [11]. In 2016, Szulzewsky et al. profiled GBM tumour-associated microglia (GAMs) identifying expression of both M1 and M2 associated genes, dependent on cell origin [12]. They identified pro-tumourigenic Osteoactivin (*GPNMB*) and Osteopontin (*SP1*) expression, supporting the role which microglia play in GBM tumour progression. Disruption of CD47-SIRPα axis using monoclonal antibodies resulted in enhanced phagocytosis of glioma cells [13] and enhanced activation of both M1 and M2 macrophage subtypes with significant shift towards the M1 (anti-tumourigenic) phenotype. This indicates that promotion of M1-microglia activation within GBM tumours represents an opportunity to enhance an anti-glioma effect.

## Immunotherapy

The immune system plays a vital role in the formation and establishment of tumours, having host-protective and tumour-promoting functions. This immune process is described as ‘cancer immunoediting’ or the ‘*three E′s’* [14]. ‘*Elimination’*, when transformed cells are successfully destroyed by a competent immune system. However, tumour cells can survive immune destruction and may enter a subsequent phase called ‘*Equilibrium’*; whereby immunoediting occurs through cell-associated antigen mutation, downregulation, deletion and/or selective survival of certain antigen negative or positive subpopulations, involving downregulation of major histocompatibility complex (MHC)—class II, increased expression of cytotoxic T lymphocyte-associated protein 4 (CTLA-4), programed cell death protein (PD-1), IL-10 and TGF-β, in addition to recruitment of regulatory T cells to dampen the immune response [15, 16]. This phase presents a major challenge to immunotherapy. ‘*Escape’*, is when immunologically edited tumours grow and present in a clinical setting, establishing an immunosuppressive tumour microenvironment where tumour infiltrating lymphocyte (TIL) activity is supressed.

**Fig. 1:**
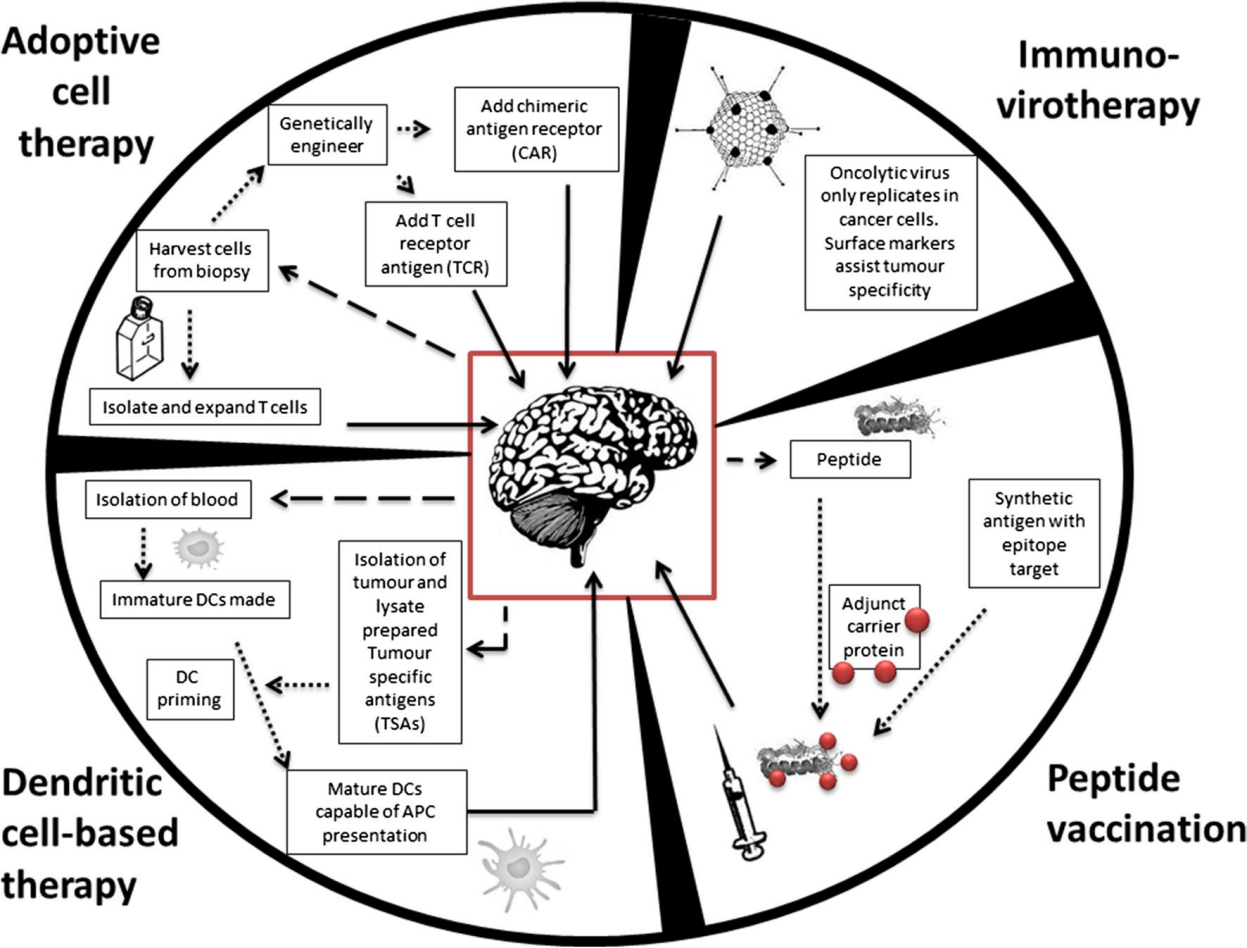
Glioblastoma immunotherapy approaches. Immunotherapy is the process by which the host immune system is modulated in an attempt to generate a tumour-targeted response. These techniques, as outlined in the graphical summary above, include adoptive cell therapy (ACT) whereby the host immune system is stimulated to elicit a response, immunovirotherapy which involves the use of oncolytic viruses which are only capable of replication within cancer cells with subsequent cell lysis. Peptide vaccinations are developed through either tumour isolated, or synthesised, peptide fragment which, when combined with carrier protein adjuvants, are then used to vaccinate the host against a particular antigen; and finally dendritic cell-based therapy whereby tumour specific antigens (TSA) and tumour associated antigens (TAAs) are used to direct a dendritic cell-prompted immune response. Several of these techniques have entered Phase III clinical trials with respect to glioblastoma treatment

Immunotherapeutic approaches can be categorised as active or passive, further summarised into several different strategies (Fig. 1). Passive immunotherapy involves the direct transfer of effector immune cells into patients to induce an anti-tumour effect; such effector cells include NK and lymphokine-activated killer (LAK) cells, but may also involve the use of antibodies or targeted toxins. Active immunotherapy aims at promoting activation of a T_h_1 immune response through tumour vaccines, non-specific immune stimulants, or cellular vaccines such as dendritic cell or tumour cell vaccines. In the following sections, we will review current research of GBM immunotherapies including the use of checkpoint inhibitors, adoptive cell therapy, immunovirotherapy, dendritic-cell-based therapy, and peptide vaccination.

*Checkpoint inhibitors* The immune system is heavily reliant upon multiple checkpoints to avoid the attack of healthy cells. Immune checkpoint proteins are surface and secreted molecules that inhibit over-activation, an aspect tumour cells often take advantage of in order to avoid detection. Checkpoint inhibitors target molecules serving as checks on the immune response, enhancing pre-existing anti-cancer immune responses. CTLA-4 and PD-1 with their corresponding ligands (CD80/CD86, PD-L1 and PD-L2) are the most extensively studied immune checkpoint proteins in cancer [17]. CTLA-4 targeting is currently in Phase III trials of recurrent GBM using Ipilimumab (Yervoy®; NCT02017717). Researchers have also assessed the beneficial therapeutic response to anti-PD-1 immunotherapy in several forms of cancer [18]. Initial studies have found that, with respect to mutational load, the greater the number of mutations present within the tumour genome, the greater the patient response to anti-PD-1 immunotherapy [19]. Based on a metastatic melanoma study by Hugo et al. [20], attenuation of the innate anti-PD-1 resistance (IPRES) transcriptional signature may help improve translational anti-PD-1 responses in cancer; although resistance mechanisms to this form of treatment have also been investigated [21]. This finding provides substantial hope for anti-PD-1 treatment in GBM which is characterised by high somatic mutations. Notably, PD-L1 expression was found to be prevalent in GBM and brain metastases, with GBM showing high PD-L1 positivity; providing promise for the use of PD-L1 inhibitors [22]. Phase II clinical trials involving two anti-PD-1 antibodies; Durvalumab (MED14736; NCT02336165) and Pembrolizumab (Keytruda®; NCT02337491) are currently underway in primary GBM patients. A randomised Phase III trial testing intravenous administration of Nivolumab (Opdivo®; NCT02017717), an anti-PD-1 antibody, in recurrent GBM patients, alone and in combination with Bevacizumab or the anti-CTLA-4 drug Ipilimumab (Yervoy®), is due for completion in 2018.

*Adoptive cell therapy (ACT)* is personalised immunotherapy where anti-tumour lymphocytes or peripheral blood mononuclear cells (PBMC) are expanded *ex vivo* and selected for efficient recognition of tumour associated antigens (TAAs). Specific TAAs can be undetectable or modestly expressed on surrounding healthy tissue, therefore serving as an attractive target for immunotherapy. ACT can exploit host cells which exhibit anti-tumour reactivity such as NK, LAKs and gamma-delta (γδ) T cells whose expansion and activation favours an anti-tumour effect [23, 24]. NK cells express a variety of activating receptors, including NKp46, NKp30, and NKp44, DNAX accessory molecule-1 (DNAM-1) and natural killer group 2, member D (NKG2D); which, upon activation, trigger NK-mediated cytotoxicity [25]. Although in vitro work is promising, there is limited data to suggest that NK cells are capable of traversing the blood–brain barrier with absence or rare detection in brain tumours [26]. Therefore induction of host immune response through alternative approaches such as vaccines or external induction of T_h_1-type response has been evaluated. LAK cells are a mixture of lymphokine-activated CD3^+^ T lymphocytes and NK cells. Phase I trials assessed LAK cells as an adjunct to biphasic antibody treatment of advanced GBM [27], which was further assessed in 2008 in solid tumours [28]. Promising Phase II trials showed, as an adjunct therapy, increased survival sufficiently warrants further evaluation in randomised trials [29], which have yet to be undertaken. Similarly, Phase I intracranial and intravenous NK cell injection showed tumour regression in a small number of patients, but with no overall survival assessment [30]. Further trials to assess improved survival have yet to be conducted.

Additionally, host cells that have been genetically modified with anti-tumour T cell receptors (TCRs) or chimeric antigen receptors (CARs) which specifically target tumour antigens (Fig. 1). TCRs on the surface of circulating T cells recognise tumour MHC-presented antigens. Depending on the antigen presentation pathway, TCRs can recognise intracellular or cross-presented antigens (Class 1, presenting to CD8+ T cells) or endocytosed antigens (Class II, presenting to CD4+ T cells). Alternatively, CARs or CAR-modified T cells are engineered receptors whereby the specificity of a monoclonal antibody is imposed onto an isolated portion of the patients T cells which, now capable of targeting tumour-specific antigens, are reinfused into the patient as targeted therapy.

ACT therapy for GBM has evolved from the use of non-specific NK and LAK cells to tumour-specific activation of the immune system, using virus specific CTLs and CAR-modified T cells. This approach has been investigated as several GBM-specific CARs have been identified [31] with current Phase I/II trials of anti-EGFRvIII CAR-T cells being held in glioma (NCT02209376 and NCT01454596). Additional Phase I studies involving allogenic CTLs expressing genetically modified T cells targeting IL13Rα2 (NCT01082926) and CAR modified CMV-specific cytotoxic lymphocytes (NCT01109095) indicate that this approach is clinically applicable, with minimal therapy related side effects and transient anti-glioma responses in a IL13Rα2-expressing GBM tumour cohort [32, 33].

*Viral immunotherapy* Another form of immunotherapy uses live viruses to carry DNA into human cells, known as viral vector vaccines. These vectors contain DNA encoding for antigens that, once expressed in the infected cells, elicit an immune response. Typically, viruses are immunogenic and can be engineered to express specific tumor antigen transcripts, resulting in an enhanced presentation of tumor antigens to the immune system. This leads to an increase in cytotoxic T lymphocytes targeting tumor cells expressing the tumor antigen encoded in the vaccine vector [34]. In addition, viruses have also been used as oncolytic agents (oncolytic virotherapy). Oncolytic viruses cannot undergo replication except in specific tumour cells, reducing off-target effects, supported through the use of specific surface markers [35]. Several viruses, including adenovirus, measles and herpes simplex, have been clinically tested as oncolytic agents [36]; however genetically-engineered adenoviruses were the first to enter clinical trials. GBM Phase I clinical trials using virotherapy include modified measles virus producing carcinoembryonic antigen (CEA; NCT00390299) and genetically engineered poliovirus PVS-RIPO (NCT01491893) recognising Necl-5, a GBM tumour antigen cell adhesion molecule [37]. GBM selective adenovirus Delta-24-RGD (DNX-2401) can infect, replicate within and destroy glioma cells [38]. Based on these promising results, researchers began a first-in-human Phase I study (NCT02197169) to assess viral capacity to replicate in gliomas. Preliminary viral immunotherapy studies use direct intratumoural administration [39, 40]; therefore efficacy of systemic administration and anti-tumour effect has yet to be assessed.

*Peptide vaccination* concerns generation of vaccines based on peptide sequences representing a tumour antigen specific target [41]. Peptide vaccinations offer the advantage of high specificity and ease of antigen-generation. Limitations include poor immunogenicity of peptides which can be circumvented through conjugation to a carrier protein such as keyhole limpet haemocyanin (KLH) or tetanus toxoid [42]. Furthermore, adjuvants have been required because soluble antigens are generally poor at driving cellular immune responses. Despite identification of several GBM targets, including specific EGFR mutations, PDGFR, PTEN and IDH1, very few have been evaluated for vaccine production. Those assessed include Rindopepimut (Rintega®, CDX-110), an EGFRvIII-based vaccine designed to target EGFRvIII-positive GBM patients, showing benefits in recurrent patients in a Phase II trial [43]. The results of the Phase II Study of Rindopepimut/GM-CSF in GBM patients (ACT III) indicated an increase in survival (NCT01480479) [44] however the Phase III (ACT IV) study was discontinued in March 2016 as the study was deemed unlikely to meet its overall survival endpoint with both the Rindopepimut and control arm performing on par with each other [45]. Additional trials using peptide-based vaccines in GBM, including those targeting IDH1 mutations, are outlined in Table 1. A lack of homogenous GBM-specific antigen expression is a central challenge for GBM targeting and peptide vaccine development, with antigen identification limited to in vitro assessment. New techniques may provide a means of identifying specific antigenic targets to enhance the endogenous immune response elicited by peptide vaccination in the GBM microenvironment. For example, Zhou et al. developed an in vivo screen involving pooled short hairpin RNA (shRNA) that were designed to target negative regulators of T cells. These targeting shRNA were then highly enriched in tumours by releasing a block on T lymphocyte proliferation upon tumour antigen recognition [46]. Such techniques may prove promising for antigen identification in further GBM vaccinations.

**Table 1:** Peptide-based vaccines for GBM therapy, data collated from https://clinicaltrials.gov

Name	Description	NCT number	Phase
Rinodepepimut (Rintega®, CDX-110)	EGFRvIII mutant targeting conjugated to keyhole limpet haemocyanin (KLH) carrier protein	NCT01480479	Randomised Phase III currently underway initial Phase III showed increased PFS and OS from point of diagnosis
HSPPC-96	An autologous heat-shock protein peptide complex-96 (HSPPC-96) vaccine for patients with recurrent GBM	NCT01814813	Randomised Phase II single arm trial showed promise however lymphopenia was noted as an adverse side effect
ERC1671	Whole GBM tumour cells and lysates from patient donors	NCT01903330	Phase II results showed great promise with reduced tumour infiltration and no noted side effects
SL-701	Synthetic multi-peptide immunotherapy consisting of three shortened peptides corresponding to IL-13, Rα2 and survivin that have been engineered with amino acid substitutions to increase immunostimulatory activity	NCT02078648	Phase I/II, currently ongoing
NeoVax	Vaccine applicable to patients with MGMT-unmethylated status	NCT02287428	Phase I currently ongoing until January 2018
ADU-623	A live-attenuated, double-deleted strain of the Gram-positive bacterium Listeria monocytogenes (Lm) encoding EGFRvIII and the cancer antigen NY-ESO-1	NCT01967758	Phase I due to complete in April 2017
GAPVAC	Several actively personalised vaccines (APVACs) tailored to the characteristics of each individual patient’s tumour	NCT02149225	Phase I due to be completed in July 2018
IMA950	Multipeptide vaccine Peptides in IMA950 comprise the following: brevican (BCAN); chondroitin sulfate proteoglycan 4 (CSPG4); fatty acid binding protein 7, brain (FABP7); insulin-like growth factor 2 mRNA binding protein 3 (IGF2BP3); neuroligin 4, X-linked (NLGN4X); neuronal cell adhesion molecule (NRCAM); protein tyrosine phosphatase, receptor-type, Z polypeptide 1 (PTPRZ1); tenascin C (TNC); Met proto-oncogene (MET); baculoviral IAP repeat-containing 5 (BIRC5); and hepatitis B virus core antigen	NCT01222221 and NCT01920191	Phase I/II completed in March 2016 reported that IMA950 plus granulocyte macrophage colony-stimulating factor (GM-CSF) was well tolerated with the primary immunogenicity endpoint of observing multi-tumour associated peptide responses in at least 30 % of patients exceeded. Further development of IMA950 is encouraged
Neoepitope-based Personalise vaccine approach	Personalised peptide vaccines	NCT02510950	Pilot study Phase 0 due for completion in March 2019
IDH R132H and PEPIDH1M	Trials to evaluate the safety and tolerability of and immune response to the IDH1 peptide vaccine in patients with IDH1R132H-mutated, WHO grade III-IV gliomas	NCT02454634 and NCT02193347	Phase I trials, due for completion in August 2018 and June 2019, respectively

*Dendritic cells (DCs)* are key antigen presenting cells (APCs) involved in the initiation of adaptive immunity. DCs take up tumour antigens, transport them to the lymph nodes, presenting via MHC Class I and II to CD8+ and CD4+ T cells and induce a tumour-specific immune response. The primary challenge in vivo is addressing the optimal mechanism through which DCs are activated, as effective vaccines should be capable of activating DCs to promote efficient T_h_1 responses and CTL [47]. As discussed previously, T_h_1 responses in addition to CTL play an important role in anti-tumour immunity so it’s essential that active immunotherapy activates DCs appropriately to provide the signals required for promoting potent cell-mediated immunity [48]. In this regard, DCs can be loaded with antigen in the presence of DC stimulating factors such as toll-like receptor (TLR) ligands to induce DC maturation [49].

In order to avoid the challenge of identifying broadly recognised tumour specific GBM antigens, DCs can be ‘primed’ using whole tumour lysates. Such methods have been employed in Phase I/II trials in GBM [50] which, when used in combination with standard clinical practise, improves patient response and survival rates. The current findings from six clinical studies [51] shows that DC immunotherapy led to a significant increase in overall and 2 year survival rates compared to standard clinical protocols with minimal toxicity. Notably, these studies have low cohort numbers, requiring further recruits before definitive conclusions can be drawn; however initial findings are promising with several Phase I and III clinical trials underway (Table 2). Recently, Mitchell et al. [52] showed that preconditioning of a DC vaccination site with an intramuscular vaccine of Td toxoid (Sanofi Aventis; Decavac; 1 Lf, 100 μl) results in a significant increase in lymph node homing and efficacy of GBM tumour antigen primed DCs in a murine model of GBM. This improvement was noted in both progression free (PF) and overall survival (OS) rates.

**Table 2:** DC-based vaccines for GBM therapy, data collated from https://clinicaltrials.gov

Name	Description	NCT number	Phase
DCVax-L	Activated monocytes loaded with antigens from the patient’s own tumour tissue	NCT00045968	Phase III, September 2016, ongoing
ICT-107		NCT01280552	Promising Phase I and II trials (Phuphanich et al. [50], http://www.imux.com 2015), Phase III trials are randomised, double-blinded, placebo-controlled to assess changes in overall survival
ICT-121	Specifically targets CD133	NCT02049489	Phase I, due for completion in November 2017, http://www.imux.com
DC vaccine	Autologous dendritic cells pulsed with lysate derived from an allogeneic glioblastoma stem-like cell line for patients with newly diagnosed or recurrent glioblastoma	NCT02010606	A phase I trial testing a dendritic cell vaccine for patients with newly diagnosed or recurrent glioblastoma, due for completion in October 2018
DC vaccine	To demonstrate that dendritic cell vaccine loaded with tumor lysate is feasible and safe in pediatric and adult subjects with relapsed high grade glioma or glioblastoma multiforme	NCT01808820	Phase I, due for completion in July 2018
CMV-specific dendritic cell vaccines	Evaluation of overcoming limited migration and enhancing cytomegalovirus-specific Dendritic Cell Vaccines with adjuvant tetanus pre-conditioning in patients with newly-diagnosed GBM	NCT02366728	Randomised Phase II, due for completion in June 2020
CMV pp65 DCs	AVeRT: Anti-PD-1 Monoclonal Antibody (Nivolumab) in combination with DC vaccines for the treatment of recurrent Grade III and Grade IV brain tumours	NCT02529072	Randomised phase I/II, due for completion in March 2019

## Imaging challenges in immunotherapy

Magnetic resonance imaging (MRI) is a medical imaging technique which is based upon the use of strong magnetic fields to detect ‘spin’ in atomic nuclei, for example the spin in hydrogen atoms from water molecules. The intensity and quality of an MRI signal is determined by two characteristics of the target tissue, the nuclear spin–lattice time (T1) and the spin–spin relaxation time (T2). Current radiographic assessment of GBM progression is based on T1-weighted (positive contrast) MRIs, however T2-weighted (negative contrast) imaging has been used for vasogenic oedema, gliosis and chemotherapy-related treatment effect visualisation [53], with radiographic response criteria stipulated under the Response Assessment in Neuro-Oncology (RANO) working group [54]. T1-weighted MRI is effective in identification of necrotic regions of brain tissue and, quite often, a contrast agent may be used to enhance imaging of additional tissue features. Primary GBM treatment involves a multimodal approach including surgical resection and radiotherapy with concomitant and adjunct chemotherapy. Three months post treatment completion, approximately 20–30 % of patients may show high contrast on MRI which may not indicate true disease progression, but rather *pseudoprogression*, caused by increased inflammation and blood brain barrier (BBB) disruption due to radiation and TMZ treatment [55]. As immunotherapy recruits the hosts immune system as a means of targeting GBM cells, inflammation occurs leading to the radiographic effect of ‘lesion’ enhancement and additional ‘lesion’ detection, suggestive of disease progression and premature cessation of immunotherapy [55]. As reviewed by Brandes et al. [56], identification of true disease progression versus pseudoprogression is a challenging pitfall in neurooncology. Given the promise of clinical trials for GBM immunotherapy, establishment of immune-related-RANO (iRANO) criteria has been undertaken. iRANO criteria define progressive disease as ‘*disease which persists beyond a determined period of time after initial radiographic evidence of progression*’ subsequent to immunotherapy completion [57], thereby providing a standardised criteria to assess the beneficial effect of immunotherapy in cancers including GBM. In addition to iRANO criteria, post-treatment diagnostics can include proton MR spectroscopic imaging (^1^H-MRSI), detecting chemical compounds and metabolites commonly detected in brain tissue including choline (Cho), creatine (Cre), lactate, lipid, and N-acetylaspartate (NAA). A recent study utilizing ^1^H-MRSI reported a 97 % success rate in retrospective differentiation between recurrent tumour and pseudoprogression and/or radiation injury, with increased Cho/NAA and Cho/Cre ratios in areas of recurrent tumour, compared with areas of lesions and normal adjacent brain tissue [58]. This, however, is not yet standard clinical practise but may, most likely, be required as immunotherapy trials increase in the future, to determine true therapeutic potential versus trial cessation.

Although immunotherapy holds great promise in terms of GBM treatment, the anti-tumour effect has yet to be proven in a translational context. Improvement in terms of overall survival rates is the primary focus of novel drug therapy mechanisms for this aggressive form of tumour. Major challenges include not only the limited number of GBM patients who are eligible to join particular clinical studies, but also a deep understanding of various regulatory and stimulatory factors in the immune system and tumour microenvironment of this highly heterogeneous tumour. Positive results from ongoing clinical trials in terms of survival benefit would ultimately result in immunotherapy becoming a standard part of the clinical treatment regime, and provide opportunities for new combination therapies to be explored.
